# New Biomaterials and Regenerative Medicine Strategies in Periodontology, Oral Surgery, Esthetic and Implant Dentistry

**DOI:** 10.1155/2015/210792

**Published:** 2015-07-27

**Authors:** David M. Dohan Ehrenfest, Hom-Lay Wang, Jean-Pierre Bernard, Gilberto Sammartino

**Affiliations:** ^1^Department of Periodontics and Oral Medicine, University of Michigan School of Dentistry, Ann Arbor, MI 48109-1078, USA; ^2^LoB5 Research Unit, School of Dentistry & Research Center for Biomineralization Disorders, Chonnam National University, Gwangju 500-757, Republic of Korea; ^3^Department of Stomatology, Implantology and Dental and Maxillofacial Radiology, School of Dental Medicine, University of Geneva, 1205 Geneva, Switzerland; ^4^Department of Oral Surgery, Faculty of Medicine, University of Naples Federico II, 80131 Naples, Italy

Periodontology, Oral Surgery, Esthetic and Implant Dentistry (the POSEID disciplines) are strongly interconnected clinical and research fields, even if they are taught as separate disciplines in most academic environments. Depending on the professional organization of each country, dental implants are often placed by periodontists or oral surgeons (and even sometimes prosthodontists), even if implant dentistry is today one of the most active independent fields of education and research by itself. Esthetic dentistry is also a general term that concerns in fact most of the Dental Arts: per definition, all dental treatments are expected to lead to an esthetic result nowadays. The separation between these dental disciplines appears sometimes quite artificial, even if each discipline has clearly its own themes and specificities. Behind this question of terminology and limits between the disciplines the question of transdisciplinarity in dental therapeutics and the need for a global conception of the dental rehabilitations rises. However, when it is related to the development of new technologies, the borders between these disciplines vanish in front of the paradigm of translational transversal research.

These POSEID dental disciplines are currently very active and are the source of development of many new techniques and technologies. It is in their domains that the highest number of innovation and publications can be found in dentistry nowadays. Implantable biomaterials were particularly investigated and have significantly evolved in the recent years: new dental implant design and surfaces [1], new bone biomaterials [2], new surgical adjuvants such as platelet concentrates [3], and so forth. The objectives of all these biomaterials and technologies are not only to replace missing or damaged tissues but also now to promote tissue regeneration [4]. This regenerative medicine approach and the many recent technological evolutions are associated with the development of new therapeutic strategies that still require to be duly evaluated and validated. We can cite, among the main current research biomaterial themes in the POSEID disciplines, the development of new biomaterials and techniques of bone grafting or bone regeneration and for periodontal and peri-implant soft tissue management [4], the development of new bone substitutes and healing membranes for periodontal and implant reconstructions [5], the development of new technologies and strategies in oral regenerative medicine and bioengineering (e.g., the use of growth factors or fibrin-based healing surgical adjuvants) [3], the development of new pharmacological technologies and strategies during periodontal treatments and oral surgery (e.g., mouthwash and oral gel), or new dental implant surfaces and design for the improvement of osseointegration [1].

All these themes are very transdisciplinary/transversal, both in their clinical impact (it concerns almost all aspects of periodontology, oral surgery, implantology, and even dentistry in general) and in their research methods (it concerns physics/biophysics, pharmacology, biology, and material sciences).

These themes are also very transversal, as they are concerning many clinical disciplines outside of dentistry, such as orthopedics/sports medicine or plastic surgery. For example, regenerative medicine strategies with platelets (Platelet-Rich Plasma (PRP) and Platelet-Rich Fibrin (PRF)) or advanced cell therapies (with various forms of stem cells) are a frequent theme in the POSEID field [4], but they are also widely developed in medical research [6]. If Leukocyte- and Platelet-Rich Fibrin (L-PRF) is sometimes perceived as a dental biomaterial (due to historical reasons and its frequent use in oral surgery and implant dentistry) [4], it is also very promising in orthopedics, sports medicine [7, 8], and regenerative strategies of the chronic ulcer wounds [9]. Transdisciplinarity is very strong in most POSEID research themes.

These research fields are also the most active translational research topics in orofacial sciences, as any research about new biomaterials or techniques requires basic sciences research, in vitro and in vivo. For example, the development of new healing growth factors based materials (such as Leukocyte- and Platelet-Rich Fibrin (L-PRF)) requires pharmacologic, biological, and tissue engineering concepts to be tested, validated, optimized, and finally redeveloped for extended applications in other fields [6, 9, 10]. On the other hand the development of new mouthwash solutions or healing gels implies biological and biophysical research (e.g., with microencapsulated bioactive molecules) for the production of new technologies and their final validation. Finally, the implantable materials are ideal examples of translational research (e.g., titanium implants or bone substitute materials) [1, 2] as they require very accurate engineering of the chemical and morphological characteristics of the materials (using physical instruments from surface and material science) [1, 11], its correlation and validation with biological behaviors and concepts [1, 12], its validation in vivo and in humans, and finally the understanding of its long-term clinical outcomes and eventual pathologies (such as associated peri-implantitis mechanisms). In this case, many parameters are integrated (surface, macrodesign, and biomechanics) [13] and have a strong impact in several domains (orthopedics particularly). It is a good example of translational transversal research.

The quantity of new biomaterials and biotechnologies with direct clinical applications in the interconnected POSEID fields is considerable and reflects very strongly this need for transdisciplinarity and translational culture [4]. However, the borders between these disciplines are often artificially maintained and the conception of these themes is still frequently lacking this global approach. For example, it can be often observed in the dental literature that teams working on implant materials (e.g., surfaces) are neglecting the material science expertise of other specialties (e.g., surface engineers) [1, 12, 14], leading sometimes to a very dental simplification of some topics and to serious approximations and flaws in the literature [1]. The same could be described in all transversal fields, such as regenerative medicine [9], platelet concentrates for surgical use [3], or bone materials [2]. The specialty-centered culture is often a big limitation for the development of these sciences and a better integration of this transdisciplinarity has to be promoted.

Finally, it is also important to notice that many review articles can be found in these fields about various aspects of these biomaterials and their clinical impact, but we are lacking specific review articles making the synthesis of new approaches in these fields and leading to international consensus. In many review articles, the conclusions are too often that data are missing. Concrete perspectives with new approaches are often lacking, while many data from other fields could allow drawing more perspectives and feeding the debates and discussions. This need for multidisciplinary debates and consensus is very strong [1, 3], but works promoting this transcollaborative culture are still scarce, even if officially promoted by most academic institutions.

As a conclusion, it is a real need nowadays to consider the research about biomaterials and biotechnologies in the interconnected fields of periodontology, oral surgery, and esthetic and implant dentistry as a whole. It is the essence of the POSEIDO Academic Consortium (Periodontology, Oral Surgery, Esthetic & Implant Dentistry Organization) to promote this transdisciplinary philosophy [15]. First, these disciplines are so tightly interconnected that research in this domain deserves often to be regrouped under such an acronym (POSEID disciplines). Second, this transdisciplinarity has to be extended to all related medical, biological, and engineering disciplines able to collaborate on these topics. Finally, the culture of translational sciences is really needed to promote development in this domain. It is expected that this POSEIDO philosophy will continue to develop and become a standard in this field.
